# The Effects of Dynamic and Static Emotional Facial Expressions of Humans and Their Avatars on the EEG: An ERP and ERD/ERS Study

**DOI:** 10.3389/fnins.2021.651044

**Published:** 2021-04-22

**Authors:** Teresa Sollfrank, Oona Kohnen, Peter Hilfiker, Lorena C. Kegel, Hennric Jokeit, Peter Brugger, Miriam L. Loertscher, Anton Rey, Dieter Mersch, Joerg Sternagel, Michel Weber, Thomas Grunwald

**Affiliations:** ^1^Swiss Epilepsy Center, Zurich, Switzerland; ^2^Department of Psychology, University of Zurich, Zurich, Switzerland; ^3^Valens Rehabilitation Centre, Valens, Switzerland; ^4^Psychiatric University Hospital Zurich, Zurich, Switzerland; ^5^Institute for the Performing Arts and Film, Zurich University of the Arts, Zurich, Switzerland; ^6^Institute for Critical Theory, Zurich University of the Arts, Zurich, Switzerland

**Keywords:** EEG, emotion, face, avatar, alpha, theta, ERP

## Abstract

This study aimed to examine whether the cortical processing of emotional faces is modulated by the computerization of face stimuli (”avatars”) in a group of 25 healthy participants. Subjects were passively viewing 128 static and dynamic facial expressions of female and male actors and their respective avatars in neutral or fearful conditions. Event-related potentials (ERPs), as well as alpha and theta event-related synchronization and desynchronization (ERD/ERS), were derived from the EEG that was recorded during the task. All ERP features, except for the very early N100, differed in their response to avatar and actor faces. Whereas the N170 showed differences only for the neutral avatar condition, later potentials (N300 and LPP) differed in both emotional conditions (neutral and fear) and the presented agents (actor and avatar). In addition, we found that the avatar faces elicited significantly stronger reactions than the actor face for theta and alpha oscillations. Especially theta EEG frequencies responded specifically to visual emotional stimulation and were revealed to be sensitive to the emotional content of the face, whereas alpha frequency was modulated by all the stimulus types. We can conclude that the computerized avatar faces affect both, ERP components and ERD/ERS and evoke neural effects that are different from the ones elicited by real faces. This was true, although the avatars were replicas of the human faces and contained similar characteristics in their expression.

## Introduction

Facial expressions of emotion play an important role in human interactions and communication. During the past few years, many studies have investigated the role of neuronal mechanisms involved in the processing of emotional faces (Krolak-Salmon et al., 2001; Balconi and Lucchiari, 2006; Moore et al., 2012). The evaluation of these mechanisms associated with the processing of facial expressions necessitates the use of valid stimuli that fully capture the facial and emotion-related information displayed in a face. However, most studies on the perception and recognition of facial expressions have used static humanoid two-dimensional pictures displaying different emotional expressions, taken from various face databases, e.g., IAPS stimuli library, Karolinska Directed Emotional Faces Database, NIMSTIM database, and many more (for an overview of databases used in emotional facial expressions research see Schindler and Bublatzky, 2020). Only very few studies applied dynamic facial expressions, which are considered to be more ecologic and therefore closer to daily life (Schacher et al., 2006; Sarkheil et al., 2013). Yet designing and generating dynamic facial stimuli poses the problem of controlling for temporal and figural properties of the face and the developing facial expression. A promising way to bypass this is the use of three-dimensional computer-generated faces, so-called avatar faces, which allow forming and systematically control important features of the facial expression. Avatars applied in research allow conducting highly standardized experiments that resemble real-life social situations (Zaki and Ochsner, 2009; de Borst and de Gelder, 2015). Hence it seems vital to investigate how humans perceive, respond to and interact with these human-like avatars. The herein presented study used videos and pictures of facial expressions (fearful, neutral) of humans and their customized avatars, to investigate whether brain activation differs in response to human and avatar facial expressions. A variety of commercial software is available to computerize human faces, most of them differ in the accuracy of displaying facial feature details. For this study, we created our own stimulus material in collaboration with the Institute for Performing Arts and Film of the ZHdK (Zurich University of the Arts). First, actors were recorded while performing fearful and neutral facial expressions. Secondly, each actor’s face was transferred into their lookalike customized avatar, created by a graphic artist to match their appearance. The motion of the facial expression was then tracked with 66 tracking points, to convey the actors’ recorded expressions onto the avatar faces.

The same stimuli material was used in a successfully published event-related fMRI work by Kegel et al. (2020). This fMRI study aimed to investigate whether there are differences in cortical and subcortical processing mechanisms when comparing human and avatar dynamic facial expressions (Kegel et al., 2020). Interestingly, their results showed no significant response difference when comparing dynamic fearful expressions between human and avatar faces for subcortical core regions involved in face and emotion perception, like the amygdala. The amygdala, together with the pulvinar and the superior colliculus is assumed to form a subcortical face processing route, particularly involved in the processing of dynamic emotional expressions (Haxby et al., 2000; Vuilleumier, 2005). Previous research has shown that in this subcortical route, the amygdala adopts a crude appraisal function processing only coarse, typically blurred information (Vuilleumier, 2005), which enables the fast interpretation of threatening environmental input, bypassing slower cortical processing (LeDoux, 2000; Adolphs, 2001; Vuilleumier et al., 2003). By providing low-spatial frequency information about coarse stimulus features like the configuration or form of a face, both, human faces and their respective avatar faces seem to generate the effect to transiently capture the viewers’ attention, which may explain the comparable amygdala responses. Another result of that fMRI study was the stronger response for fearful human compared to avatar faces for cortical structures such as the anterior and posterior superior temporal sulcus (STS), and the inferior frontal gyrus (IFG). Previous research has shown that the STS and the IFG represent the dorsal face processing pathway, specialized and sensitive for dynamic facial features (Duchaine and Yovel, 2015). On the one hand, there were technical limitations to the avatar faces that could be associated with the found difference, as subtle motions and skin texture were not detectable. On the other hand, Sarkheil et al. (2013) concluded that even when using advanced motion tracking and visualization techniques, the artificial facial motion of avatars leads to differences in the activation of these regions when compared to human facial motion. Taken together Kegel et al. (2020) concluded, that dynamic avatar expressions evoke fast amygdala responses comparable to those of humans, but the artificial facial motion of those avatars lead to differences in slower cortical process mechanisms.

To complement these very detailed structural findings, the herein presented EEG study was conducted to get a more detailed insight into the temporal resolution of facial emotional encoding. It is worth to mention, that the herein presented EEG study was done independent of the fMRI measurements and therefore a new set of participants were recruited. Since both ERPs and ERD/ERS may reflect different neurophysiological processes (Pfurtscheller, 1997; Hajcak et al., 2010), the present study uses both methods. In comparison, ERPs reflect evoked activity that are both time- and phase-locked to the event, whereas internal or external events modulate the induced oscillatory rhythm, resulting in a time-, but not necessarily phase-locked activity (Gomarus et al., 2006). Studies on event-related potentials (ERP) show that human facial expressions are probably recognized and differentiated within the primary 200–250 ms after their presentation (Bentin et al., 1996; Leppänen et al., 2007; Balconi and Pozzoli, 2009). Eimer and Holmes (2007) acknowledged that emotional faces were found to trigger an increased ERP positivity relative to neutral faces. In addition to ERP, brain oscillations are a powerful tool to investigate the cognitive processes related to emotion comprehension. The differentiation of facial expression induces significant change, mainly, event-related desynchronization (ERD) in alpha and event-related synchronization (ERS) in theta oscillations (Güntekin and Başar, 2007, 2014; Moore et al., 2012). Difficulties in facial emotion perception are found in several diseases like epilepsy (Hlobil et al., 2008; Zhao et al., 2014) and schizophrenia (Kohler et al., 2000; Trémeau, 2006). Only very few studies are dealing with the effect of avatar faces on the emotional face processing mechanisms in ERPs. With respect to the effect of artificial faces on ERPs, several components are of interest: The N100 is a negative potential peaking at around 100 ms after stimulus onset, and previous studies have demonstrated that the N100 is significantly affected by facial perception and sensitive to facial expression (Eimer and Holmes, 2007; Jessen et al., 2012). The N170 is a component occurring 120–200 ms after stimulus onset and is prominent over temporo-parietal leads (Bentin et al., 1996). This ERP component is associated with the analysis of facial configuration, free from the influence of sex, age, and race (Eimer, 2000; Ishizu et al., 2008) and can be elicited by schematic drawings (Sagiv and Bentin, 2001; Carmel and Bentin, 2002) and isolated facial features, such as the eyes (Bentin et al., 1996). A study by Schindler et al. (2017) used six face-stylization levels varying from abstract to realistic and could show that the N170 showed a u-shaped modulation, with stronger reactions toward the most abstract and to the most realistic ones, compared to medium-stylized faces. Another ERP component of interest in face processing is the N300 (Barrett and Rugg, 1990; Bentin and Deouell, 2000; Eimer, 2000). This component peaks within 250–350 ms after stimulus onset. It can be measured over the left and right hemispheres and over central, frontal, and parietal regions and has been found to differentiate between familiar and unfamiliar stimuli. More specifically, familiar faces, such as those of celebrities or politicians, typically elicit responses that are larger in amplitude than those of N300s to unfamiliar faces (Scott et al., 2005). A study by Debruille et al. (2012) showed that dummy faces lead to greater amplitudes than those of real persons. They claimed that the greater N300 amplitudes index the greater inhibition that is needed after the stronger activations induced by this stimulus. Late positive potentials (LPP) occurring at around 400–600 ms are a reliable electrophysiological index of emotional perception in humans. Emotional faces and increased face realism prompt larger LPPs (Schupp et al., 2000; Pastor et al., 2008; Bublatzky et al., 2014) than those to neutral faces, thus reflecting an activity increase in the visual and parietal cortex. These late potentials seem to increase linearly with face realism, thus reflecting an increase of activity in visual and parietal cortex for the more realistic faces (Schindler et al., 2017).

Frequency bands that have acquired the attention in the differentiation of facial expression research are the theta (4–8 Hz) and alpha (8–10 Hz) bands, both of which are sensitive to variations in task demands (Balconi and Lucchiari, 2006; Başar et al., 2006; Güntekin and Başar, 2007). For the processing of a dynamic facial expression, it has been shown that emotional faces tend to elicit stronger theta synchronization than neutral faces (Aftanas et al., 2001, 2002; Başar et al., 2008; Balconi and Pozzoli, 2009), but not much is known about the effect of watching emotional avatar faces. A study by Urgen et al. (2013) showed that the observation of action performed by a robot results in greater frontal theta activity compared to action performed by androids and humans, whereas the latter two did not differ from each other. Thus, they concluded that frontal theta appears to be sensitive to visual appearance and suggested that artificial agents appearance may evoke greater memory processing demands for the observer. Different aspects of alpha oscillations are considered to play a part during emotional processing and visual attention-involving, like frontal alpha asymmetry (Allen et al., 2004; Davidson, 2004; Pönkänen and Hietanen, 2012; Goodman et al., 2013) or ERD over occipital, temporal and posterior areas (Aftanas et al., 2001; Sato et al., 2004; Pineda, 2005; Güntekin and Başar, 2007). On the functional level, alpha frequencies are considered to represent cortical activation of the corresponding region of the underlying cerebral cortex (Klimesch, 1999). It was shown that the observation of movements and facial pain mimic expressed by an artificial agent induced significant attenuation in the power of mu oscillations but without any difference between the agents (robot, android, and human) (Urgen et al., 2013; Joyal et al., 2018). A decrease in alpha power for positive and negative or arousing emotions in comparison with neutral stimuli was detected on several electrode locations (Sarlo et al., 2005; Balconi and Pozzoli, 2009), but so far to our knowledge, no studies were performed with fearful dynamic facial expression in avatars.

Taken together, although avatars offer a promising approach to study facial expressions, to date, the evoked brain activation patterns by avatar faces have received little detailed study. To our knowledge, this is the first study in which the method of ERD/ERS is used together with the analysis of ERPs at the same time during the presentation of dynamic and static avatar and human fearful and neutral facial expressions. In the present study, we asked whether both ERPs and cortical oscillatory dynamics in a group of healthy adults are affected by the type of face agent transmitting the emotional information. Based on the results of the fMRI study by Kegel et al. (2020) we expected to find comparable outcomes for our event-related and oscillatory EEG study. We presumed that early and late ERP components are differentially sensitive to actor and avatar faces. We anticipated that fast subcortical processes do not affect early potentials, whereas slower cortical processes lead to variations in later ERP components. Additionally, we expected to find effects on the power of both theta and alpha oscillations. We assumed that dynamic emotional facial expression should result in a more pronounced theta ERS, independent of the agent, whereas increased attentional demands of encoding artificial avatar faces should be reflected in a more pronounced alpha oscillation desynchronization.

## Materials and Methods

### Participants

Twenty-five healthy participants aged between 24 and 62 (17 female; *M*_*age*_ = 40.5 years; *SD*_*age*_ 11.3 years) participated in the study and all reported normal vision (no color blindness, no uncorrected vision disorder) and no history of neurological disorders. The study was approved by the local ethics committee, and participants gave their written informed consent in line with the Declaration of Helsinki and received compensation for travel expenses.

### Stimuli and Design

**FIGURE 1:**
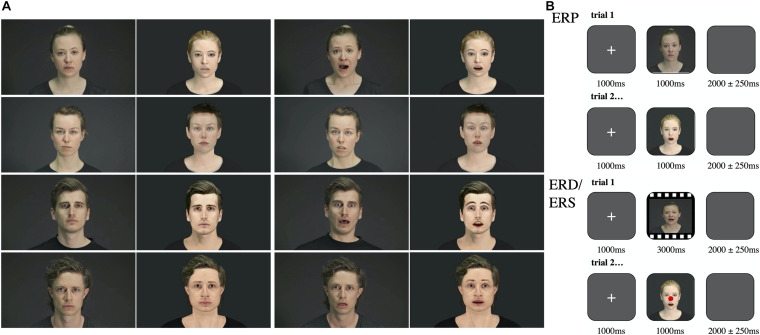
Stimuli and procedure. **(A)** Depiction of the female actors on the upper left and the male actors on the lower left during neutral and fearful expression. Their respective avatars are shown on next to them. **(B)** Top: Depiction of the procedure during the ERP trials: After a fixation cross, a picture (1 s) showing either a human or an avatar facial expression was presented. Participants were instructed to passively watch the pictures and to respond with a button press for indicating a male or female face. Each picture was separated with a blanc screen. Bottom: Depiction of the procedure during the ERD/ERS trials: After a fixation cross, a video (3 s) showing either a human or an avatar facial expression was presented. Participants were instructed to passively watch the videos and to respond with a button press during control trials (red dot). Each video was separated with a blanc screen.

We used a set of videos that had been developed for the present study in a three-step process in cooperation with the Zurich University of the Arts: (i) Fearful and neutral human facial expressions were recorded from four actors (two female, two male). (ii) For each actor, a customized avatar was created by a graphic artist (Pinterac SARL, France) to match their appearance (see Figure 1). (iii) By motion tracking with 66 tracking points (FaceRig©, Holotech Studios SRL, Romania), the actors’ recorded expressions were conveyed onto the avatar faces (see study by Kegel et al., 2020). For simplification, we will use the term “avatar” and “actor” as a synonym for the face, as no body movements were shown neither for the actor nor the avatar. For each actor and each avatar, eight fearful and eight neutral videos were created, resulting in a total of 128 videos, each lasting three seconds. For the ERP study, one screenshot was taken from each video at timepoint 2:00 s, resulting in 128 pictures. This resulted in four stimuli conditions: Agent (actor, avatar) and emotion (neutral, fear). Participants were instructed to sit still, passively viewing the presented 128 brief film clips (3 s) and pictures (1 s) of faces of actors or avatars showing either neutral or fearful expression.

#### ERP Task

The pictures were presented in pseudo-randomized order with an interval of 2000 ± 250 ms of the blank screen followed by a fixation cross (1000 ms). To keep our subjects engaged we asked them to indicate on each trial whether the presented face was female or male by pressing one of two buttons accordingly.

#### ERD/ERS Task

The clips were presented in pseudo-randomized order with an interval of 2000 ± 250 ms of the blank screen followed by a fixation cross (1000 ms). To keep subjects engaged we mixed 16 control trials with pictures taken from the videos modified with a red dot in the middle of the face in between the clips and asked the subjects to push a button when control pictures appeared. All subjects performed well in the control tasks and seemed to be engaged through the whole experiment.

### Electrophysiological Recording and Analysis

Participants sat in a comfortable chair in a sound-attenuated room with dimmed lights facing a portable computer screen in 1-m distance. For EEG recordings 21 sintered Ag/AgCl scalp electrodes were placed according to the international 10–20 system, referenced to the linked mastoids using an EEG cap (Multicap, Neurolite). Continuous EEG signals were recorded from a NicoletOne C64 amplifier (Natus, United States). The data, including a rectangular trigger signal, were sampled at 256 Hz. All data and signal processing was performed in asa^TM^ (ANT Neuro), through custom-written routines in Matlab (Math Work Inc., United States) and the Matlab Plugin EEGlab (version 2020.0). Offline data were re-referenced to the average of both mastoids (A1 + A2) and then filtered with a forward 0.16 Hz high-pass and a zero-phase 32 Hz low-pass filter. A natural gradient logistic infomax independent component analysis (ICA) was performed on the data (the runica algorithm; EEGLAB toolbox, version b, Delorme and Makeig, 2004) to decompose the EEG mixed signals into their underlying neural and artifactual components. The ADJUST algorithm (v1.1; Mognon et al., 2011) was used to identify and remove artifacts, such as eye and muscle movements. This left a total of 25 participants with an average of 123.42 trials (*SD* = 2.36) overall (neutral avatar, *M* = 30.98; neutral actor, *M* = 31.02; fear avatar, *M* = 30.55; fear actor = 30.87) for the ERD/ERS task and 125.50 trials (*SD* = 2.57) overall (neutral avatar, *M* = 31.86; neutral actor, *M* = 32.65; fear avatar, *M* = 30.56; fear actor = 30.43) for the ERP task. There were no significant differences in the number of kept trials between the four stimuli categories.

#### Event-Related Potential

For each participant, averaged ERP waveforms were computed across trials separately for each condition. Filtered data were segmented from 200 ms before stimulus onset until 1000 ms after stimulus presentation. The 200 ms before stimulus onset were used for baseline correction. Within these time intervals, the mean amplitude values were determined. For statistical analyses, four ERP components known to be sensitive for facial perception were defined (N100, N170, N300, LPP). After ERP waveform inspection, those electrodes with the most prominent amplitudes were selected for further analysis. This resulted in the following electrode clusters: N100 (F3, Fz, F4, F7, F8), N170 (T5, T6), N300 (F3, Fz, F4, C3, Cz, C4, T3, T4), LPP (C3, Cz, C4, P3, Pz, P4). Time windows of interest were chosen based on visual inspection of our collapsed conditions ERP plots and in accordance to similar previous studies (Bentin et al., 1996; Zhang et al., 2008; Luo et al., 2010; Choi et al., 2014). The intervals were ranging from 120 to 180 ms for the N100, from 120 to 230 ms for the N170, from 250 to 350 ms for the N300, and from 400 to 600 ms for the LPP.

#### ERD/ERS

The data for ERD analysis were epoched and averaged for each stimulus condition separately ranging from 3000 ms before video onset to 4000 ms after video onset and were time-locked to the onset of the video clips. In agreement with the commonly accepted frequency ranges for theta and alpha, we defined the theta frequency band as ranging from 4 to 8 Hz and the alpha band from 8 to 12 Hz. ERD/ERS was calculated as the percentage of increase or decrease in power relative to the baseline interval (–2000 to –1000 ms pre-stimulus) before clip onset (Pfurtscheller and Aranibar, 1977). To perform the time-frequency analysis we used the event-related spectral perturbation (ERSP) plot proposed by Makeig (1993). The ERSP plot was computed using the Matlab function newtimef.m, which was one of the time-frequency decomposition functions (Delorme and Makeig, 2004). The FFT-estimated results (Hanning window tapering) were shown in log spectral differences from 200 ms baseline (in dB), with the red and blue indicating power increase and decrease, respectively. Time windows of interest for each frequency band were determined from the mean spectrographic image (ERSP) across all conditions and are in line with previous studies (Aftanas et al., 2001; Onoda et al., 2007; Urgen et al., 2013). From the ERSPs we determined specific time windows for statistical analyses for alpha (500–2000 ms) and theta (140–400 ms) post-stimulus and calculated the absolute power with respect to the baseline (EEGLAB toolbox plug Darbeliai V2015.11.08.1). With regard to spatial sampling points, electrodes were collapsed into electrode clusters. This procedure resulted in six regional means for each hemisphere: anterior temporal (AT); frontal (F); central (C); temporal (T); parietal (P); and occipital (O). The average ERD/ERS values across the respective electrode sites were calculated for these regional means for each specific time interval and each stimulus condition.

### Statistical Analysis

Statistical analyses were performed on SPSS Statistics 25.0 (IBM, Somers, United States).

#### Event-Related Potential

For each component, the mean amplitude was measured for the predefined time window and selected electrode channels. The data were entered into an Emotion (2: neutral/left) × Agent (2: actor/avatar) repeated measures ANOVA. We also modeled Hemisphere (left, right) for all components to explore any modulation that may be specific to the hemisphere side. These analyses are not reported since they did not reveal any side-specific effects or interactions, and the effects reported below for the repeated measures ANOVA did not change. For the analysis of variance, degrees of freedom were Greenhouse–Geisser corrected for violations of the sphericity assumption. The probability of a Type I error was maintained at 0.05. *Post hoc* testing of significant main effects was conducted using the Bonferroni method. Partial eta-squared (η^2^_*p*_) was estimated to describe effect sizes. Here, η^2^_*p*_ = 0.2 describes a small, η^2^_*p*_ = 0.5 a medium and η^2^_*p*_ = 0.8 a large effect (Lakens, 2013).

#### ERD/ERS

For theta, the mean power in the time window of the theta increase (140–400 ms after stimulus onset) was extracted for each condition (agent and emotion combination) for all regional means (AT, F, C, P, O, T). Only the regional means that showed a significant increase of the theta power were selected for further analysis and the data were entered into an Emotion (2: neutral/left) × Agent (2: actor/avatar) repeated measures ANOVA. Mean alpha power in the time window of the alpha attenuation (500–2000 ms after stimulus onset) was extracted for each condition (agent and emotion combination) for all regional means (AT, F, C, P, O, T). Because all regional means displayed significant attenuation of the alpha power, for the sake of clarity we decided to condense the electrode positions into even broader means: frontal (AT and F), central (C), parieto-occipital (P and O), and temporal (T). We entered the dependent variable of alpha mean power into a three-way ANOVA using the following repeated factors: Emotion (2: neutral/left) × Agent (2: actor/avatar) × Regional Mean (4: frontal, central, parieto-occipital, temporal). We also modeled Hemisphere (left, right) for both frequency bands to explore any modulation that may be specific to the hemisphere side, only taking the regional means with significant attenuation/increase into account. These analyses are not reported since they did not reveal any side-specific effects or interactions, and the effects reported below for the repeated measures ANOVA did not change. For the analysis of variance, degrees of freedom were Greenhouse–Geisser corrected for violations of the sphericity assumption. The probability of a Type I error was maintained at 0.05. *Post hoc* testing of significant main effects was conducted using the Bonferroni method. Partial eta-squared (η^2^_*p*_) for ANOVA was estimated to describe effect sizes. Here, η^2^_*p*_ = 0.02 describes a small, η^2^_*p*_ = 0.13 a medium and η^2^_*p*_ = 0.26 a large effect (Lakens, 2013).

## Results

### Event-Related Potentials

#### N100

Regarding the N100 amplitudes, no main effects of agent [*F*_1_,_24_ = 0.165, *p* = 0.686, η^2^_*p*_ = 0.001] and emotion [*F*_1_,_24_ = 1.186, *p* = 0.278, η^2^_*p*_ = 0.009] and no interaction effect [*F*_2_,_48_ = 3.671, *p* = 0.278, η^2^_*p*_ = 0.028] were detected.

#### N170

**FIGURE 2:**
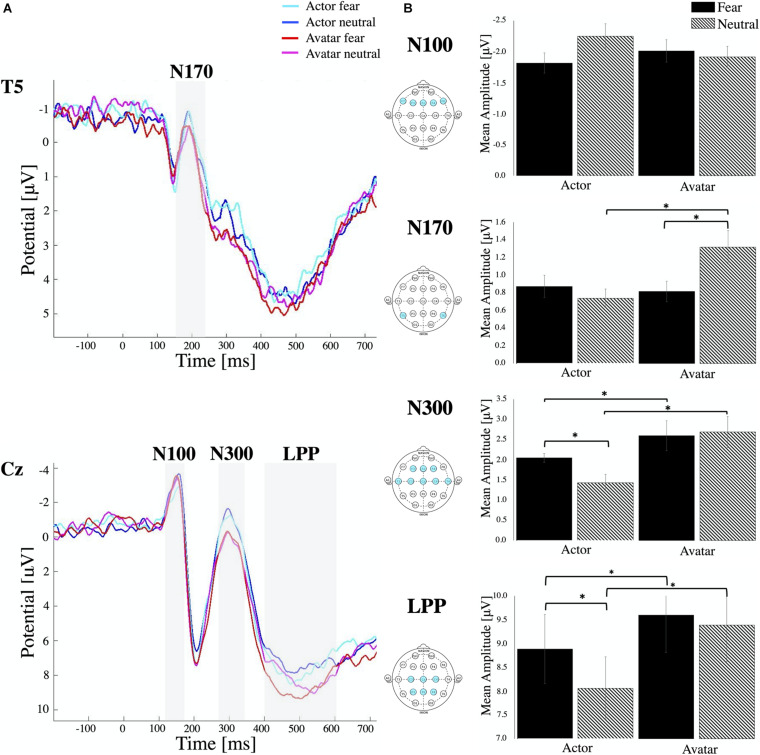
ERP components. **(A)** Stimulus locked ERP response elicited by actor and avatar faces at electrode position T5 and Cz averaged across all participants. The ERP components N100, N170, N300 and LPP are highlighted in the grey shaded regions. **(B)** Mean amplitude values for each ERP component averaged for respective channel locations. Error bars indicate the standard error of the mean (^∗^ all *p* < 0.01). Note that, while negative-going, the N170 and N300 peak is still in the positive range. Therefore, smaller bars represent higher amplitudes.

For N170, the mean amplitude was measured within a window that best captured this component (120–230 ms after stimulus onset) at temporal electrodes (T5, T6) (Figure 2). Repeated measures ANOVA analysis showed a significant interaction of Agent × Emotion [*F*_2_,_48_ = 5.535, *p* < 0.02, η^2^_*p*_ = 0.103] without any significant main effects. *Post hoc* paired samples *t*-test reflected more negative amplitudes for fearful than neutral avatar faces [*t* = –2.826, *p* < 0.007] but a more pronounced N170 for actor faces in the neutral condition compared to the avatar agent [*t* = –2.485, *p* < 0.02]. Comparisons of the N170 revealed no significant amplitude differences across hemispheres.

#### N300

For the N300 component, the average amplitude response within 250–350 ms after stimulus onset was measured from the left and right hemispheres and from central, temporal and frontal regions (Figure 2). Statistical analysis revealed a main effect for Emotion [*F*_1_,_24_ = 6.36, *p* < 0.01, η^2^_*p*_ = 0.024] and Agent [*F*_1_,_24_ = 51.44, *p* < 0.00, η^2^_*p*_ = 0.210] with a significant interaction effect of Agent × Emotion [*F*_2_,_48_ = 8.26, *p* < 0.00, η^2^_*p*_ = 0.049]. *Post hoc* comparisons (paired samples *t*-test) indicated that the N300 was more pronounced in neutral condition compared to fear, which was only significant for the agent actor [*t* = 3.55, *p* < 0.00]. There was no difference in the emotional condition for the avatar, but we could find a significant decrease in N300 amplitudes to both fearful [*t* = –3.54, *p* < 0.00] and neutral faces of avatars [*t* = –6.93, *p* < 0.00] compared to those to the faces of actors. There were no significant differences in N300 amplitude between electrode positions on left and right hemisphere and between central, frontal and temporal electrode locations.

#### Late Positive Potentials

The time window between 400 and 600 ms was selected for examining LPPs and in compliance with literature, we also found the greatest positivity for the central and parietal electrode (Figure 2). Repeated measures ANOVA revealed a main effect for Emotion [*F*_1_,_24_ = 40.37, *p* < 0.00, η^2^_*p*_ = 0.097] and Agent [*F*_1_,_24_ = 43.70, *p* < 0.00, η^2^_*p*_ = 0.227] with a significant interaction effect of Agent × Emotion [*F*_2_,_48_ = 14.14, *p* < 0.01, η^2^_*p*_ = 0.044]. *Post hoc* comparisons (paired samples *t*-test) indicated that LPPs were more pronounced in fear condition compared to neutral, which was only significant for the agent actor [*t* = 5.35, *p* < 0.00]. There was no difference in the emotional condition for the avatar, but we could detect a significant increase in LPP for both emotional conditions [fear *t* = –3.34, *p* < 0.00, and neutral *t* = –7.72, *p* < 0.00] for the avatar compared to the actor. In contrast to other studies, we couldn’t detect any significant effect of the hemisphere of electrode location. There were no significant differences between central and parietal electrode locations.

### ERD/ERS

**FIGURE 3:**
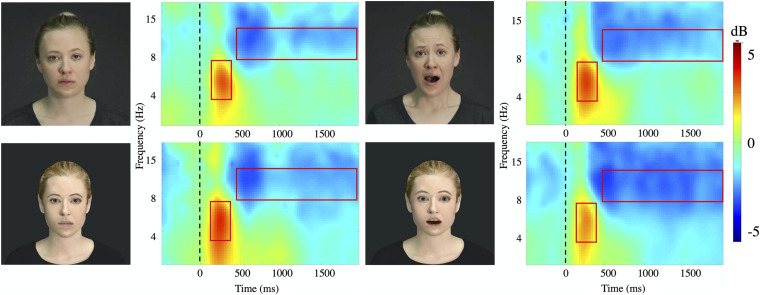
Time-frequency plots. Time frequency plots for the four stimuli conditions (Actor: fear/neutral; Avatar, fear/neutral) at channel Pz averaged for all subjects. The frequency axis is log scaled. The zero point on the time axis indicates the onset of the video presentation. The red squares represent the time window (*x* axis: theta 140–400 ms; alpha 500–2000 ms) and frequency band (*y* axis: theta 4–8 Hz; alpha 8–12 Hz) taken for further analysis.

Both, faces of actors and avatars with either neutral or fearful expression lead to a strong ERS in theta (4–8 Hz) followed by a significant attenuation in alpha (8–12 Hz) after stimulus onset (Figure 3). All types of stimuli elicited comparable amounts of significant ERD (alpha 76%) and ERS (theta 76%) for all trials. There was no significant main effect of Hemisphere.

#### Theta Oscillations

**FIGURE 4:**
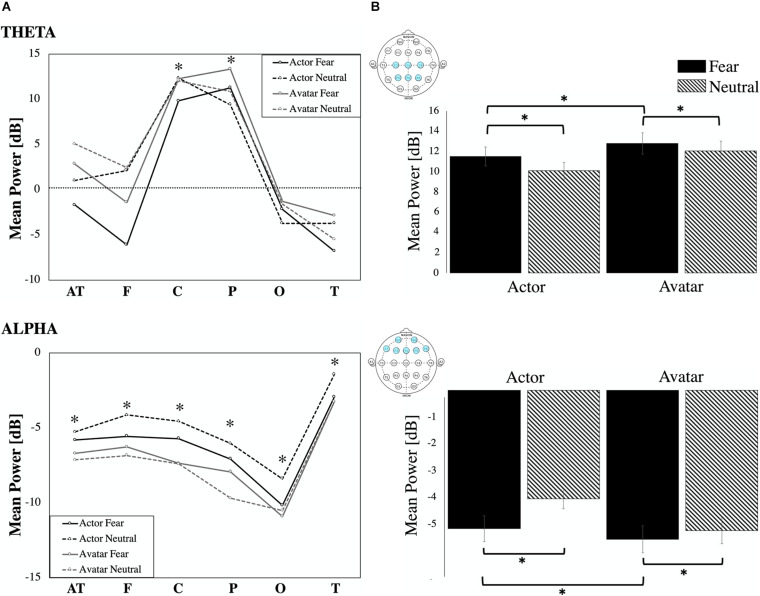
ERD/ERS. **(A)** ERD/ERS by location: Event-related synchronization and desynchronization in percentage for the theta (4–8 Hz) and alpha frequency band (8–12 Hz) for the six regional means (AT, F, C, P, O T). Statistically significant increase and decrease power are marked (^∗^ all *p*’s < 0.01). **(B)** Power (in dB) in theta and alpha frequency for the four video conditions (actor: fear/neutral; avatar: fear/neutral) averaged for respective channel locations. Error bars indicate the standard error of the mean.

Relative to the pre-stimulus baseline, the theta band (4–8 Hz) showed significant ERS with maximum amplitudes at 140–400 ms (Figure 4A) over the central (*Z* = –8.50, *p* < 0.00, η^2^_*p*_ = 0.210) and parietal (*Z* = –10.04, *p* < 0.00, η^2^_*p*_ = 0.323) areas for the two agents: actor (percentage of significant ERS trials: fear: C 45.76%, P 63.38%, neutral: C 43.24%, P 55.58%) and avatar (percentage of significant ERS trials: fear: C 44.93%, P 60.86%, neutral: C 58.22%, P 75.36%). Our main comparison of interest, a 2 (Agent) × 2 (Emotion) repeated measures ANOVA at central and parietal electrodes revealed a significant main effect of Emotion [*F*_1_,_24_ = 23.55, *p* < 0.00, η^2^_*p*_ = 0.139] (Figure 4B). *Post hoc* paired samples *t*-test comparisons indicated that theta oscillations were significantly greater for the fear condition compared with the neutral condition for both agents [actor *t* = 1.97, *p* < 0.05; avatar *t* = 2.68, *p* < 0.03]. The effect of Agent [*F*_1_,_24_ = 13.08, *p* < 0.00, η^2^_*p*_ = 0.082] was also significant. *Post hoc* comparisons (paired samples *t*-test) showed higher mean power values for the avatar compared to the actor agent, which was only significant for the fear condition [*t* = –4.62, *p* < 0.00]. There was no significant Agent × Emotion interaction effect and there were no significant hemisphere effects (left/right) for the specific electrode position with regard to the emotion displayed.

#### Alpha Oscillations

Because all regional means (AT, F, C, P, O, T) showed a significant ERD relative to the pre-stimulus baseline in the alpha band (8–12 Hz) with maximum amplitudes at 500–2000 ms (Figure 4A) post-stimulus, we condensed the electrode positions into even broader means: frontal (AT and F), central (C), parieto-occipital (P and O), and temporal (T). We entered the dependent variable of alpha mean power into a three-way ANOVA using the following repeated factors: Emotion (2: neutral/left) × Agent (2: actor/avatar) × Electrode Position (4: frontal, central, parieto-occipital, temporal) (Figure 4B). The repeated measures ANOVA showed a significant main effect for Agent [*F*_1_,_24_ = 15.34, *p* < 0.00, η^2^_*p*_ = 0.029]. Parietal sites showed highest ERD values. Temporal sites were less activated than frontal [*t* = –7.40, *p* < 0.00], central [*t* = –5.33, *p* < 0.00] and parietal [*t* = –6.92, *p* < 0.00] ones. *T*-test *post hoc* comparisons revealed significantly higher desynchronization for the avatar compared to the actor in the neutral condition for frontal [*t* = 2.57, *p* < 0.01], central [*t* = 2.38, *p* < 0.02], and temporal [*t* = 2.65, *p* < 0.01] areas. There were no significant hemisphere effects (left/right) for the specific electrode areas with regard to the emotion displayed.

## Discussion

In the present study, we aimed to investigate whether facial static and dynamic emotional information of faces of human actors and their avatars elicit similar or different electrocortical responses. Except for the very early N100, all ERP features selected for analysis differed in their response to avatar and actor faces. Whereas the N170 showed differences only for the neutral avatar condition, later potentials (N300 and LPP) differed in both emotional conditions (neutral and fear) and the presented agents (actor and avatar). These results are in line with the findings of the fMRI study (Kegel et al., 2020), in which fearful human expressions elicited stronger responses than the fearful avatar expressions in cortical structures such as the STS, and the IFG, but not for subcortical structures like the amygdala. In addition, we found that the avatar faces elicited significantly stronger reactions than the actor face for theta and alpha oscillations. Especially theta EEG frequencies responded specifically to visual emotional stimulation and revealed to be sensitive to the emotional content of the face, whereas alpha frequency was modulated by all the stimulus types.

### Event-Related Potentials

Except for the N100, all ERP features analyzed in the present study distinguished between avatar and actor faces. Each of these components is thought to be related to a different aspects of the process of facial expression, from fast neural processing of visual features to slower emotion-specific discrimination and higher-level cognitive processing (Luo et al., 2010). The very early ERP component peaking at around 100 ms post-stimulus is thought to be mainly involved in the subcortical processing of visual information. Previous studies have demonstrated that the early anterior N100 is significantly affected by affect in the early phase of perception and attention processing (Batty and Taylor, 2003; Eimer and Holmes, 2003). It is modulated by physical features of visual stimuli (Eimer et al., 2008), especially during face perception (Pegna et al., 2004; Palermo and Rhodes, 2007). A study by Luo et al. (2010) found that not only facial detection but also the emotional content of a face can influence the N100 amplitude. Fearful faces differed from neutral faces, suggesting that there may exist a fast scanning process for emotional discrimination before completing more detailed perceptual processes. Our results show a clear early negative peak around 100 ms in anterior areas but without any significant differences in amplitude between these stimuli, independent of the agent or emotional condition. This is in line with the results of the fMRI study Kegel et al. (2020). No response difference was detectable when comparing dynamic fearful expressions between human and avatar faces for subcortical core regions involved in face and emotion perception, like the amygdala. The presented avatar faces were replicas of actor faces with the same dominant face characters, like hair color and style, eyes, etc. Thus, differences between faces of actors and avatars may have been not distinct enough to be detected during this very early stage of visual processing. It is also assumed that by providing low-spatial frequency information about coarse stimulus features like the configuration or form of a face, both, human faces and their respective avatar faces seem to generate the effect to transiently capture the viewers’ attention, what may explain the comparable N100 responses.

The N170 is a dominant component elicited during the later stage of visual information encoding. It reflects high-level structural processes (Bentin et al., 1996) and is typically more pronounced in response to faces, including schematic ones (Sagiv and Bentin, 2001), than to any other category of objects, such as animals or cars (Carmel and Bentin, 2002; George et al., 2005; Schindler et al., 2017). Studies suggest that the N170 is sensitive to the realism of faces, and pictures of robots and androids elicited smaller and delayed N170 amplitudes than pictures of humans (Dubal et al., 2011; Urgen et al., 2012). A study by Schindler et al. (2017) revealed an interesting relation between amplitude and the level of stylization of a face, with stronger reactions toward both most abstract and most realistic faces compared to medium-stylized faces. Several findings indicated that the N170 is also modulated by emotional content, with larger amplitude for emotional than for neutral faces (Batty and Taylor, 2003; Pourtois et al., 2005; Zhang et al., 2012). Moreover, emotional effects at the N170 can be found for faces of medium stylization (Japee et al., 2009) and even for robots with rather schematic faces (Pessoa et al., 2005). In our study, neutral avatar faces were perceived differently than all other stimuli and led to significant attenuation in the N170 amplitude. It seems like this early component reflects initial perceptual awareness of visual stimuli and the neutral avatar face was perceived as the least “face like” one. The lack of N170 increase for fearful human faces is a bit unexpected, but can be caused by different factors: The herein chosen time window of 90 ms seems relatively broad, compared to other studies reporting usually narrower time windows of around 50 ms (for a review see Schindler and Bublatzky, 2020). This time window was chosen after visual inspection of the ERP plots where we could see a high variance between subjects with regard to the peak timing. One explanation for that phenomenon could be the rather big age group difference in our study (24–62 years). It has been shown that age can have delaying and reducing effects on the N170 peak (Daniel and Bentin, 2012) and our rather heterogenic participant group could have a possible effect on the peak distribution. By broadening the time window we wanted to make sure to cover the N170, but are aware of the threat of measuring maybe also other potentials like the EPN (early posterior negativity), happening over the same parieto-occipital areas. Another limiting factor poses the choice of electrodes taken into analysis. It is still discussed where to measure the N170 most effectively and we have just started to begin to understand the time and spatial resolution of the N170 component. Recommendations exist to measure the N170 at electrodes mainly in occipitotemporal (Jacques et al., 2007) and temporo-parietal areas (Bentin et al., 1996). In this study, we had a relatively sparse electrode distribution and couldn’t cover the PO7/PO8 area sufficiently. We found the N170 to be most pronounced on temporal sites and couldn’t find any reliable effects at occipital electrodes (O1/O2). For getting more robust information on the N170 spatial characteristics it makes sense to include a higher density EEG for upcoming studies. It is also worth to mention, that our trials did not include any control trials with non-facial objects as this was not part of the scope of this work, but we would assume that the response to the neutral avatar face would still be more pronounced than those to any non-face object. Interestingly we could find a difference in the emotional condition only for the avatar but not for the actor agents. This could suggest that the difference of emotional expression between fear and response in our actor stimuli material was not as obvious as to be represented in that early ERP component. As it is not quite clear whether the fearful faces were not “fearful” enough or whether the neutral faces had already some kind of emotional content in it, it would make sense in further studies to include a third emotional condition like “happy” to elaborate on that issue.

During the later stage of the encoding of visual information, we can see differences not only between the agents but also between the emotional content. The N300 amplitude is known to reflect the further evaluation of information related to the affective valence of a face, can index processes involved in the recognition of visual objects, and is showing its highest amplitudes in response to stimuli rated as more activating in the arousal dimension (Carretié et al., 1997a,b). A study by Urgen et al. (2012) showed less pronounced N300 negativity for Android compared to Human and Robot movements, possibly indicating a modulation by the (in)congruence of form and motion. Debruille et al. (2012) could find more negative N300 for a dummy face than those to a real face photo but stated that the dummy was already emotionally charged by its frightening look. In the present study, we can see more pronounced negativity for the actor agent compared to the avatar in both emotional conditions. It seems like the actor’s faces are more engaging and higher in arousal. We could say, therefore, that N300 reflects mainly neural reactions associated with the arousal charge of visual stimuli. With respect to the valence dimension, our results indicate that activating real face stimuli evoked the highest N300 amplitudes at frontal locations. In the later course, we can see that the neutral avatar face is still perceived differently than the actor, but the difference between the two emotional avatar conditions vanishes. The N300 largely reflects the dimensionality of affective valence (Carretie et al., 2001), and some studies found that emotional facial expression can elicit enhanced negative N300 (Schutter et al., 2004; Bar-Haim et al., 2005). Indeed we found a difference in the N300 amplitudes regarding the emotional condition for the actor agent, but in contrast, the decrease was in the neutral condition. This effect was not visible for the avatars and an even opposite effect can be found for the LPP. It is unclear whether the neutral stimulus contains somehow an arousing component but further experiments should include more stimuli in the low-arousal extreme of the arousal dimension to get a more detailed insight.

In general, an increased LPP amplitude is related to the intrinsic significance of picture content (Donchin and Coles, 1998) and modulated by a general increase in arousal and negative valence across the stimulus conditions (Schupp et al., 2000; Leite et al., 2012). Our results show a clear increase for avatar faces, whereas a difference in amplitudes for the emotional aspect can only be distinguished for the human actor face. This absence of emotional effects for the avatar condition is in accordance with similar studies showing that attention can distract from emotional information (Eimer and Holmes, 2007; daSilva et al., 2016). The LPP is also highly dependent on the type of experimental task. More pronounced effects are measurable when attention is directed to the emotional expression or to emotionally relevant features of a face (Rellecke et al., 2012). The LPP is sensitive to the emotional content of various stimulus types, including faces and even emotional scenes (Schupp et al., 2004; Wieser et al., 2010; Schindler et al., 2017). A study by Thom et al. (2014) found that LPP components show significantly enhanced modulation by presented emotional scenes, relative to face stimuli, suggesting that viewing emotional scenes results in a more pronounced emotional experience and the difference is not due to emotional engagement but to the valence of the stimuli. A study by Cheetham et al. (2015) shows a consistent pattern in the relationship between lesser degrees of human likeness and greater arousal and more negative valence. The data show a significantly more positive-going LPP for the avatar compared with the human category faces, and higher LPP amplitudes have been reported for emotional than for neutral stimuli (Flaisch et al., 2011; Bublatzky et al., 2014), again reinforcing the role of the LPP of reflecting higher-order evaluation and episodic memory encoding (Schupp et al., 2006).

### ERD/ERS

So far only very few studies have addressed effects of avatars on our cortical excitability, especially when exhibiting emotional facial expressions. Two oscillations seem to be associated with the encoding of facial expression and facial features, the theta and the alpha frequency band, and the extent of theta synchronization and alpha desynchronization seem to be related to different aspects of memory performance (Girges et al., 2014).

According to Klimesch (1999), the theta oscillation seems to respond selectively to the encoding of new information into episodic memory. It also reflects memory processes such as retrieval from long-term memory and encoding into long-term memory (Kahana et al., 2001; Klimesch et al., 2010). In the process of encoding facial information, it seems to be also important how “human-like” the presented face looks like. Urgen et al. (2013) proposed that theta power would decrease as a function of the human likeness of the observed agent. They hypothesized that the observation of a non-human robot would elicit a more pronounced theta activity than the observation of a human. On the other hand, they expected the human-like appearance of the agent would facilitate access to semantic representations related to human action. Their results suggest that observation of relatively unfamiliar agents could result in greater memory processing demands (Zion-Golumbic et al., 2010; Atienza et al., 2011) compared to a more human agent. We can strengthen this theory with our results, but differences between the presented agents were only measurable in the emotional fear condition, not during the presentation of the neutral stimuli. This would support the hypothesis that theta is more sensitive to and involved in emotional perception but not in face feature perception *per se*. Theta synchronization can be also a marker of emotion processing. Previous studies have shown that a difference in the amount of increase of spectral power in theta frequency is discernable between emotional and neutral stimuli, but it is less obvious for discriminating the valence of emotional stimuli (Aftanas et al., 2001, 2004; Başar et al., 2006; Knyazev et al., 2008). This implies that theta synchronization may not be associated with a particular emotion. Rather, it may indicate complex operations involved in the processing of emotional information.

The intensity of alpha desynchronization reflects general task demands and attentional processes. It has been shown that it is topographically widespread over the entire scalp and the more difficult or relevant a task, the more pronounced the amount of alpha suppression (Klimesch, 1999). In our study the observation of human as well as the avatar faces resulted in robust and significant attenuations in the alpha power over all electrodes, peaking at centro-parieto sites. Our results are in line with previous findings suggesting that an activity increase within this region may reflect greater attentional demands to natural facial motion (Girges et al., 2014). A difference in the amount of alpha suppression evoked by the two agents was discernable in the neutral condition significant on frontal, central, and temporal sites, but not for parieto-occipital sites. Mixed results can be found in other studies. Urgen et al. (2013) indeed found significant desynchronization in the alpha band over sensorimotor areas, frontal and parietal while presenting videos of humans, androids, and robots performing smaller movements but could not detect any differences between responses to the presented agents. They stated that particularly the mu rhythm over sensorimotor areas seems not to be selective only for other humans, but showed robust and significant modulations also during the observation of robot actions. This data also suggests that the suppression of the mu rhythm does not seem to be modulated by early stages of action processing as there were no differences detectable for the visual appearance or movement kinematics of the agents. Another study by Saygin et al. (2012) also found no difference between human and robot actions in the premotor cortex. Instead the parietal cortex seemed to be sensitive to the match of the motion and appearance of the agent, evident by significant differences in response to the Android. Girges et al. (2014) suggesting that the missing differentiation for the parieto-occipital areas suggest that early visual processing remains unaffected by manipulation paradigms. In our study, the difference between responses to the different agents was discernable in the neutral condition. We take this finding to suggest that alpha is mainly involved in general attentional demands during facial information processing and can be modulated by all stimulus types, without being specific for emotional content. This can be underlined by the finding that there were no differences between responses to the emotional stimuli.

The stimuli used for the present study were unique in the way that static images and dynamic clips of actors were transformed into their matching avatars. The avatars included all main facial characteristics like general symmetry, skin color, hair-style, etc., but without too many details on texture like wrinkles or marks. Motion tracking was used to transfer the mimic of the actor onto the artificial avatar face, and some of the smaller movements that were captured got lost during that process and made the avatar appearance more simple. The goal was to create an artificial face close enough to the actor template, but not too detailed to be mistaken for real faces, to avoid any uncanny valley effect. This effect refers to unease and discomfort feelings that people encounter when looking at increasingly realistic virtual humans (Mori et al., 2012). As already discussed, the fMRI study by Kegel et al. (2020) used the same dynamic stimuli for their experiments. Participants were asked afterward to rate the presented dynamic faces for the subjective intensity and as expected, they rated the fearful expression as more intense than the neutral, for both actors and avatars. Fearful human expressions were judged as more intense than fearful avatar expressions without any differences between the neutral condition. Taken together Kegel et al. (2020) concluded, that dynamic avatar expressions evoke early amygdala responses to emotional stimuli that are comparable to those of humans, but the artificial facial motion of those avatars is likely to be processed differently compared to their human counterpart. Our findings for the ERP and ERD/ERS experiments are in line with the fMRI results as we could demonstrate that static and dynamic avatar faces elicit cognitive processes that are different from the ones elicited by real human faces, and the quality of emotional content does not seem to be identical for the two agents.

Overall, we demonstrated that artificial avatar faces elicit neural effects that are different from the ones elicited by real faces. This was true, although the avatars were replicas of the human faces and contained similar characteristics in their expression. Human interactions with actors and avatars generate different internal responses. However, we believe that despite these potential limitations the use of avatars still offers benefits for further experiments on face perception and emotional encoding and also for the various commercial and public applications. We recommend that one has to take into account, that depending on the overall goal the optimal design of the avatar will differ.

## Data Availability Statement

The raw data supporting the conclusions of this article will be made available by the authors, without undue reservation.

## Ethics Statement

The studies involving human participants were reviewed and approved by Kanton Zürich, Kantonale Ethikkomission. The patients/participants provided their written informed consent to participate in this study.

## Author Contributions

TS, DM, AR, HJ, and PB conceived and planned the experiments. ML, MW, and JS contributed to the creation of the stimulus material. TS, OK, and LK carried out the experiments. TS, OK, TG, and PH contributed to the interpretation of the results. TS took the lead in writing the manuscript. All authors provided critical feedback and helped shape the research, analysis, and manuscript.

## Conflict of Interest

The authors declare that the research was conducted in the absence of any commercial or financial relationships that could be construed as a potential conflict of interest.

## References

[B1] Adolphs R. (2001). The neurobiology of social cognition. *Curr. Opin. Neurobiol.* 11 231–239. 10.1016/S0959-4388(00)00202-6 11301245

[B2] Aftanas L. I Reva N. V. Varlamov A. A. Pavlov S. V. Makhnev V. P. (2004). Analysis of evoked EEG synchronization and desynchronization in conditions of emotional activation in humans: temporal and topographic characteristics. *Neurosci. Behav. Physiol.* 34 859–867. 10.1023/B:NEAB.0000038139.39812.eb 15587817

[B3] Aftanas L. I. Varlamov A. A. Pavlov S. V. Makhnev V. P. Reva N. V. (2001). Affective picture processing: event-related synchronization within individually defined human theta band is modulated by valence dimension. *Neurosci. Lett.* 303 115–118. 10.1016/S0304-3940(01)01703-7 11311506

[B4] Aftanas L. I. Varlamov A. A. Pavlov S. V. Makhnev V. P. Reva N. V. (2002). Time-dependent cortical asymmetries induced by emotional arousal: EEG analysis of event-related synchronization and desynchronization in individually defined frequency bands. *Int. J. Psychophysiol.* 44 67–82. 10.1016/S0167-8760(01)00194-5 11852158

[B5] Allen J. B. Coan J. A. Nazarian M. (2004). Issues and assumptions on the road from raw signals to metrics of frontal EEG asymmetry in emotion. *Biol. Psychol.* 67 183–218. 10.1016/j.biopsycho.2004.03.007 15130531

[B6] Atienza M. Crespo-Garcia M. Cantero J. L. (2011). Semantic congruence enhances memory of episodic associations: role of theta oscillations. *J. Cogn. Neurosci.* 23 75–90. 10.1162/jocn.2009.21358 19925185

[B7] Balconi M. Lucchiari C. (2006). EEG correlates (Event-related desynchronization) of emotional face elaboration: a temporal analysis. *Neurosci. Lett.* 392 118–123. 10.1016/j.neulet.2005.09.004 16202519

[B8] Balconi M. Pozzoli U. (2009). Arousal effect on emotional face comprehension. frequency band changes in different time intervals. *Physiol. Behav.* 97 455–462. 10.1016/j.physbeh.2009.03.023 19341748

[B9] Bar-Haim Y. Lamy D. Glickman S. (2005). Attentional bias in anxiety: a behavioral and ERP study. *Brain Cogn.* 59 11–22. 10.1016/j.bandc.2005.03.005 15919145

[B10] Barrett S. E. Rugg M. D. (1990). Event-Related Potentials and the Semantic Matching of Pictures. *Brain Cogn.* 14 201–212. 10.1016/0278-2626(90)90029-N 2285513

[B11] Başar E. Güntekin B. Öniz A. (2006). Chapter 4 principles of oscillatory brain dynamics and a treatise of recognition of faces and facial expressions. *Prog. Brain Res.* 159 43–62. 10.1016/S0079-6123(06)59004-1 17071223

[B12] Başar E. Schmiedt-Fehr C. Öniz A. Başar-Eroğlu C. (2008). Brain oscillations evoked by the face of a loved person. *Brain Res.* 1214 105–115. 10.1016/j.brainres.2008.03.042 18471805

[B13] Batty M. Taylor M. J. (2003). Early processing of the six basic facial emotional expressions. *Cogn. Brain Res.* 17 613–620. 10.1016/S0926-6410(03)00174-5 14561449

[B14] Bentin S. Allison T. Puce A. Perez E. McCarthy G. (1996). Electrophysiological studies of face perception in humans. *J. Cogn. Neurosci.* 8 551–565. 10.1162/jocn.1996.8.6.551 20740065 PMC2927138

[B15] Bentin S. Deouell L. Y. (2000). Structural encoding and identification in face processing: ERP evidence for separate mechanisms. *Cogn. Neuropsychol.* 17 35–55. 10.1080/026432900380472 20945170

[B16] Bublatzky F. Gerdes A. White A. J. Riemer M. Alpers G. W. (2014). Social and emotional relevance in face processing: happy faces of future interaction partners enhance the late positive potential. *Front. Hum. Neurosci.* 8:493. 10.3389/fnhum.2014.00493 25076881 PMC4100576

[B17] Carmel D. Bentin S. (2002). Domain specificity versus expertise: factors influencing distinct processing of faces. *Cognition* 83 1–29. 10.1016/S0010-0277(01)00162-7 11814484

[B18] Carretie C. Mercado F. Tapia M. Hinojosa J. E. (2001). Emotion, attention, and the ‘Negativity Bias’, studied through event-related potentials. *Int. J. Psychophysiol.* 41 75–85. 11239699 10.1016/s0167-8760(00)00195-1

[B19] Carretié L. Iglesias J. García T. (1997a). A study on the emotional processing of visual stimuli through event- related potentials. *Brain Cogn.* 34 207–217. 10.1006/brcg.1997.0895 9220086

[B20] Carretié L. Iglesias J. García T. Ballesteros M. (1997b). N300, P300 and the emotional processing of visual stimuli. *Electroencephalogr. Clin. Neurophysiol.* 103 298–303. 10.1016/S0013-4694(96)96565-7 9277632

[B21] Cheetham M. Wu L. Pauli P. Jancke L. (2015). Arousal, valence, and the uncanny valley: psychophysiological and self-report findings. *Front. Psychol.* 6:981. 10.3389/fpsyg.2015.00981 26236260 PMC4502535

[B22] Choi D. Nishimura T. Motoi M. Egashira Y. Matsumoto R. Watanuki S. (2014). Effect of empathy trait on attention to various facial expressions: evidence from N170 and late positive potential (LPP). *J. Physiol. Anthropol.* 33:18. 10.1186/1880-6805-33-18 24975115 PMC4083863

[B23] Daniel S. Bentin S. (2012). Age-related changes in processing faces from detection to identification: ERP Evidence. *Neurobiol. Aging* 33:206.e1–28. 10.1016/j.neurobiolaging.2010.09.001.Age-related PMC3025306 20961658

[B24] daSilva E. B. Crager K. Geisler D. Newbern P. Orem B. Puce A. (2016). Something to sink your teeth into: the presence of teeth augments ERPs to mouth expressions. *NeuroImage* 127 227–241. 10.1016/j.neuroimage.2015.12.020 26706446

[B25] Davidson R. J. (2004). What does the prefrontal cortex ‘Do’ in affect: perspectives on frontal EEG asymmetry research. *Biol. Psychol.* 67 219–234. 10.1016/j.biopsycho.2004.03.008 15130532

[B26] de Borst A. W. de Gelder B. (2015). Is it the real deal? Perception of virtual characters versus humans: an affective cognitive neuroscience perspective. *Front. Psychol.* 6:1576. 10.3389/fpsyg.2015.00576 26029133 PMC4428060

[B27] Debruille J. B. Brodeur M. B. Porras C. F. (2012). N300 and social affordances: a study with a real person and a dummy as stimuli. *PLoS One* 7:e0047922. 10.1371/journal.pone.0047922 23118908 PMC3485319

[B28] Delorme A. Makeig S. (2004). EEGLAB: an open source toolbox for analysis of single-trial EEG dynamics including independent component analysis. *J. Neurosci. Methods* 134 9–21. 10.1016/j.jneumeth.2003.10.009 15102499

[B29] Donchin E. Coles M. (1998). Context updating and the P300. *Behav. Brain Sci.* 21 152–154. 10.1017/S0140525X98230950

[B30] Dubal S. Foucher A. Jouvent R. Nadel J. (2011). Human brain spots emotion in non humanoid robots. *Soc. Cogn. Affect. Neurosci.* 6 90–97. 10.1093/scan/nsq019 20194513 PMC3023084

[B31] Duchaine B. Yovel G. (2015). A revised neural framework for face processing. *Annu. Rev. Vis. Sci.* 1 393–416. 10.1146/annurev-vision-082114-35518 28532371

[B32] Eimer M. (2000). The face-specific N170 component reflects late stages in the structural encoding of faces. *NeuroReport* 11 2319–2324. 10.1097/00001756-200007140-00050 10923693

[B33] Eimer M. Holmes A. (2003). The Role of spatial attention in the processing of facial expression: an ERP study of rapid brain responses to six basic emotions. *Cogn. Affect. Behav. Neurosci.* 3 97–110. 12943325 10.3758/cabn.3.2.97

[B34] Eimer M. Holmes A. (2007). Event-related brain potential correlates of emotional face processing. *Neuropsychologia* 45 15–31. 10.1016/j.neuropsychologia.2006.04.022 16797614 PMC2383989

[B35] Eimer M. Kiss M. Holmes A. (2008). Links between rapid ERP responses to fearful faces and conscious awareness. *J. Neuropsychol.* 2(Pt 1), 165–181. 10.1348/174866407X245411 19330049 PMC2661068

[B36] Flaisch T. Häcker F. Renner B. Schupp H. T. (2011). Emotion and the processing of symbolic gestures: an event-related brain potential study. *Soc. Cogn. Affect. Neurosci.* 6 109–118. 10.1093/scan/nsq022 20212003 PMC3023087

[B37] George N. Jemel B. Fiori N. Chaby L. Renault B. (2005). Electrophysiological correlates of facial decision: insights from upright and upside-down mooney-face perception. *Cogn. Brain Res.* 24 663–673. 10.1016/j.cogbrainres.2005.03.017 15890502

[B38] Girges C. Wright M. J. Spencer J. V. O’Brien J. (2014). Event-related alpha suppression in response to facial motion. *PLoS One* 9:e0089382. 10.1371/journal.pone.0089382 24586735 PMC3929715

[B39] Gomarus H. K. Althaus M. Wijers A. A. Minderaa R. B. (2006). The effects of memory load and stimulus relevance on the EEG during a visual selective memory search task: an ERP and ERD/ERS study. *Clin. Neurophysiol.* 117 871–884. 10.1016/j.clinph.2005.12.008 16442346

[B40] Goodman R. N. Rietschel J. C. Lo L. C. Costanzo M. E. Hatfield B. D. (2013). Stress, emotion regulation and cognitive performance: the predictive contributions of trait and state relative frontal EEG alpha asymmetry. *Int. J. Psychophysiol.* 87 115–123. 10.1016/j.ijpsycho.2012.09.008 23022494

[B41] Güntekin B. Başar E. (2007). Emotional face expressions are differentiated with brain oscillations. *Int. J. Psychophysiol.* 64 91–100. 10.1016/j.ijpsycho.2006.07.003 17156875

[B42] Güntekin B. Başar E. (2014). A review of brain oscillations in perception of faces and emotional pictures. *Neuropsychologia* 58 33–51. 10.1016/j.neuropsychologia.2014.03.014 24709570

[B43] Hajcak G. Macnamara A. Olvet D. M. (2010). Event-related potentials, emotion, and emotion regulation: an integrative review. *Dev. Neuropsychol.* 35 129–155. 10.1080/87565640903526504 20390599

[B44] Haxby J. V. Hoffman E. A. Gobbini M. A. (2000). The distributed human neural system for face perception. *Trends Cogn. Sci.* 4 223–233. 10.1016/S1364-6613(00)01482-0 10827445

[B45] Hlobil U. Rathore C. Alexander A. Sarma S. Radhakrishnan K. (2008). Impaired facial emotion recognition in patients with mesial temporal lobe epilepsy associated with hippocampal sclerosis (MTLE-HS): side and age at onset matters. *Epilepsy Res.* 80 150–157. 10.1016/j.eplepsyres.2008.03.018 18468867

[B46] Ishizu T. Ayabe T. Kojima S. (2008). Configurational factors in the perception of faces and non-facial objects: an ERP study. *Int. J. Neurosci.* 118 955–966. 10.1080/00207450701769398 18569153

[B47] Jacques C. d’Arripe O. Rossion B. (2007). The time course of the inversion effect during individual face discrimination. *J. Vis.* 7:3. 10.1167/7.8.3 17685810

[B48] Japee S. Crocker L. Carver F. Pessoa L. Ungerleider L. G. (2009). Individual Differences in Valence Modulation of Face-Selective M170 Response. *Emotion* 9 59–69. 10.1037/a0014487 19186917 PMC2767163

[B49] Jessen S. Obleser J. Kotz S. A. (2012). How bodies and voices interact in early emotion perception. *PLoS One* 7:e0036070. 10.1371/journal.pone.0036070 22558332 PMC3340409

[B50] Joyal C. C. Neveu S. M. Boukhalfi T. Jackson P. L. Renaud P. (2018). Suppression of sensorimotor alpha power associated with pain expressed by an avatar: a preliminary EEG study. *Front. Hum. Neurosci*. 12:273. 10.3389/fnhum.2018.00273 30038564 PMC6046452

[B51] Kahana M. J. Seelig D. Madsen J. R. (2001). Theta returns. *Curr. Opin. Neurobiol.* 11 739–744. 10.1016/S0959-4388(01)00278-1 11741027

[B52] Kegel L. C. Brugger P. Frühholz S. Grunwald T. Hilfiker P. Kohnen O. (2020). Dynamic human and avatar facial expressions elicit differential brain responses. *Soc. Cogn. Affect. Neurosci.* 15 303–317. 10.1093/scan/nsaa039 32232359 PMC7235958

[B53] Klimesch W. (1999). EEG alpha and theta oscillations reflect cognitive and memory performance: a review and analysis. *Brain Res. Rev.* 29 169–195. 10.1016/S0165-0173(98)00056-3 10209231

[B54] Klimesch W. Freunberger R. Sauseng P. (2010). Oscillatory mechanisms of process binding in memory. *Neurosci. Biobehav. Rev.* 34 1002–1014. 10.1016/j.neubiorev.2009.10.004 19837109

[B55] Knyazev G. G. Bocharov A. V. Levin E. A. Savostyanov A. N. Slobodskoj-Plusnin J. Y. (2008). Anxiety and oscillatory responses to emotional facial expressions. *Brain Res.* 1227 174–188. 10.1016/j.brainres.2008.06.108 18639533

[B56] Kohler C. G. Bilker W. Hagendoorn M. Gur R. E. Gur R. C. (2000). Emotion recognition deficit in schizophrenia: association with symptomatology and cognition. *Biol. Psychiatry* 48 127–136. 10.1016/S0006-3223(00)00847-7 10903409

[B57] Krolak-Salmon P. Fischer C. Vighetto A. Mauguière F. (2001). Processing of facial emotional expression: spatio-temporal data as assessed by scalp event-related potentials. *Eur. J. Neurosci.* 13 987–994. 10.1046/j.0953-816X.2001.01454.x 11264671

[B58] Lakens D. (2013). Calculating and reporting effect sizes to facilitate cumulative science: a practical primer for t-tests and ANOVAs. *Front. Psychol.* 4:863. 10.3389/fpsyg.2013.00863 24324449 PMC3840331

[B59] LeDoux J. (2000). Emotion circuits in the brain. *Annu. Rev. Neurosci.* 23 155–184. 10.1146/annurev.neuro.23.1.155 10845062

[B60] Leite J. Carvalho S. Galdo-Alvarez S. Alves J. Sampaio A. Gonçalves O. F. (2012). Affective picture modulation: valence, arousal, attention allocation and motivational significance. *Int. J. Psychophysiol.* 83 375–381. 10.1016/j.ijpsycho.2011.12.005 22226675

[B61] Leppänen J. M. Moulson M. C. Vogel-Farley V. K. Nelson C. A. (2007). An ERP study of emotional face processing in the adult and infant brain. *Child Dev.* 78 232–245. 17328702 10.1111/j.1467-8624.2007.00994.x PMC2976653

[B62] Luo W. Feng W. He W. Yi Wang N. Jia Luo Y. (2010). Three stages of facial expression processing: ERP study with rapid serial visual presentation. *NeuroImage* 49 1857–1867. 10.1016/j.neuroimage.2009.09.018 19770052 PMC3794431

[B63] Makeig S. (1993). Auditory event-related dynamics of the EEG spectrum and effects of exposure to tones. *Electroencephalogr. Clin. Neurophysiol* 86 183–193. 7682932 10.1016/0013-4694(93)90110-h

[B64] Mognon A. Jovicich J. Bruzzone L. Buiatti M. (2011). ADJUST: an automatic EEG artifact detector based on the joint use of spatial and temporal features. *Psychophysiology* 48 229–240. 10.1111/j.1469-8986.2010.01061.x 20636297

[B65] Moore A. Gorodnitsky I. Pineda J. (2012). EEG Mu component responses to viewing emotional faces. *Behav. Brain Res.* 226 309–316. 10.1016/j.bbr.2011.07.048 21835208

[B66] Mori M. MacDorman K. F. Kageki N. (2012). “The uncanny valley,” in *Proceedings of the IEEE Robotics and Automation Magazine*, Vol. 19 (New York, NY: IEEE), 98–100. 10.1109/MRA.2012.2192811

[B67] Onoda K. Okamoto Y. Shishida K. Hashizume A. Ueda K. Yamashita H. (2007). Anticipation of affective images and event-related desynchronization (ERD) of alpha activity: an MEG study. *Brain Res.* 1151 134–141. 10.1016/j.brainres.2007.03.026 17408598

[B68] Palermo R. Rhodes G. (2007). Are you always on my mind? A review of how face perception and attention interact. *Neuropsychologia* 45 75–92. 10.1016/j.neuropsychologia.2006.04.025 16797607

[B69] Pastor M. C. Bradley M. M. Löw A. Versace F. Moltó J. Lang P. J. (2008). Affective picture perception: emotion, context, and the late positive potential. *Brain Res.* 1189 145–151. 10.1016/j.brainres.2007.10.072 18068150 PMC2993239

[B70] Pegna A. J. Khateb A. Michel C. M. Landis T. (2004). Visual recognition of faces, objects, and words using degraded stimuli: where and when it occurs. *Hum. Brain Mapp.* 22 300–311. 10.1002/hbm.20039 15202108 PMC6872030

[B71] Pessoa L. Japee S. Ungerleider L. G. (2005). Visual awareness and the detection of fearful faces. *Emotion* 5 243–247. 10.1037/1528-3542.5.2.243 15982091

[B72] Pfurtscheller G. (1997). EEG event-related desynchronization (ERD) and synchronization (ERS). *Electroencephalogr. Clin. Neurophysiol.* 1:26. 10.1016/0013-4694(77)90092-x 72657

[B73] Pfurtscheller G. Aranibar A. (1977). Event-related cortical desynchronization detected by power measurements of scalp EEG. *Electroencephalogr. Clin. Neurophysiol.* 42 817–826. 10.1016/0013-4694(77)90235-8 67933

[B74] Pineda J. A. (2005). The functional significance of Mu rhythms: translating ‘Seeing’ and ‘Hearing’ into ‘Doing’. *Brain Res. Rev.* 50 57–68. 10.1016/j.brainresrev.2005.04.005 15925412

[B75] Pönkänen L. M. Hietanen J. K. (2012). Eye contact with neutral and smiling faces: effects on autonomic responses and frontal EEG asymmetry. *Front. Hum. Neurosci.* 6:112. 10.3389/fnhum.2012.00122 22586387 PMC3343319

[B76] Pourtois G. Thut G. Grave De Peralta R. Michel C. Vuilleumier P. (2005). Two electrophysiological stages of spatial orienting towards fearful faces: early temporo-parietal activation preceding gain control in extrastriate visual cortex. *NeuroImage* 26 149–163. 10.1016/j.neuroimage.2005.01.015 15862215

[B77] Rellecke J. Sommer W. Schacht A. (2012). Does processing of emotional facial expressions depend on intention? Time-resolved evidence from event-related brain potentials. *Biol. Psychol.* 90 23–32. 10.1016/j.biopsycho.2012.02.002 22361274

[B78] Sagiv N. Bentin S. (2001). Structural encoding of human and schematic faces: holistic and part-based processes. *J. Cogn. Neurosci.* 13 937–951. 10.1162/089892901753165854 11595097

[B79] Sarkheil P. Goebe R. Schneider F. Mathiak K. (2013). Emotion unfolded by motion: a role for parietal lobe in decoding dynamic facial expressions. *Soc. Cogn. Affect. Neurosci.* 8 950–957. 10.1093/scan/nss092 22962061 PMC3831559

[B80] Sarlo M. Buodo G. Poli S. Palomba D. (2005). Changes in EEG alpha power to different disgust elicitors: the specificity of mutilations. *Neurosci. Lett.* 382 291–296. 10.1016/j.neulet.2005.03.037 15925105

[B81] Sato W. Yoshikawa S. Kochiyama T. Matsumura M. (2004). The amygdala processes the emotional significance of facial expressions: an FMRI investigation using the interaction between expression and face direction. *NeuroImage* 22 1006–1013. 10.1016/j.neuroimage.2004.02.030 15193632

[B82] Saygin A. P. Chaminade T. Ishiguro H. Driver J. Frith C. (2012). The thing that should not be: predictive coding and the uncanny valley in perceiving human and humanoid robot actions. *Soc. Cogn. Affect. Neurosci.* 7 413–422. 10.1093/scan/nsr025 21515639 PMC3324571

[B83] Schacher M. Winkler R. Grunwald T. Kraemer G. Kurthen M. Reed V. (2006). Mesial temporal lobe epilepsy impairs advanced social cognition. *Epilepsia* 47 2141–2146. 10.1111/j.1528-1167.2006.00857.x 17201715

[B84] Schindler S. Bublatzky F. (2020). Attention and emotion: an integrative review of emotional face processing as a function of attention. *Cortex* 130 362–386. 10.1016/j.cortex.2020.06.010 32745728

[B85] Schindler S. Zell E. Botsch M. Kissler J. (2017). Differential effects of face-realism and emotion on event-related brain potentials and their implications for the uncanny valley theory. *Sci. Rep.* 7:45003. 10.1038/srep45003 28332557 PMC5362933

[B86] Schupp H. T. Cuthbert B. N. Bradley M. M. Cacioppo J. T. Tiffany I. Lang P. J. (2000). Affective picture processing: the late positive potential is modulated by motivational relevance. *Psychophysiology* 37 257–261. 10.1017/S0048577200001530 10731776

[B87] Schupp H. T. Flaisch T. Stockburger J. Junghöfer M. (2006). Emotion and attention: event-related brain potential studies. *Prog. Brain Res.* 156 31–51. 10.1016/S0079-6123(06)56002-9 17015073

[B88] Schupp H. T. Junghöfer M. Weike A. I. Hamm A. O. (2004). The selective processing of briefly presented affective pictures: an ERP analysis. *Psychophysiology* 41 441–449. 10.1111/j.1469-8986.2004.00174.x 15102130

[B89] Schutter D. J. De Haan E. H. F. Van Honk J. (2004). Functionally dissociated aspects in anterior and posterior electrocortical processing of facial threat. *Int. J. Psychophysiol.* 53 29–36. 10.1016/j.ijpsycho.2004.01.003 15172133

[B90] Scott L. S. Shannon R. W. Nelson C. A. (2005). Behavioral and electrophysiological evidence of species-specific face processing. *Cogn. Affect. Behav. Neurosci.* 5 405–416. 10.3758/CABN.5.4.405 16541811 PMC9661448

[B91] Thom N. Knight J. Dishman R. Sabatinelli D. Johnson D. C. Clementz B. (2014). Emotional scenes elicit more pronounced self-reported emotional experience and greater EPN and LPP modulation when compared to emotional faces. *Cogn. Affect. Behav. Neurosci.* 14 849–860. 10.3758/s13415-013-0225-z 24374599

[B92] Trémeau F. (2006). A review of emotion deficits in schizophrenia. *Dialogues Clin. Neurosci.* 8 59–70. 10.31887/dcns.2006.8.1/ftremeau 16640115 PMC3181757

[B93] Urgen B. A. Plank M. Ishiguro H. Poizner H. Saygin A. P. (2012). “Temporal dynamics of action perception: the role of biological appearance and motion kinematics,” in *Preceedings of the 34th Annual Conference of Cognitive Science Society*, (Portland, OR: Cognitive Science Society), 2469–2474.

[B94] Urgen B. A. Plank M. Ishiguro H. Poizner H. Saygin A. P. (2013). EEG theta and mu oscillations during perception of human and robot actions. *Front. Neurorobot.* 7:19. 10.3389/fnbot.2013.00019 24348375 PMC3826547

[B95] Vuilleumier P. (2005). How brains beware: neural mechanisms of emotional attention. *Trends Cogn. Sci.* 9 585–594. 10.1016/j.tics.2005.10.011 16289871

[B96] Vuilleumier P. Armony J. L. Driver J. Dolan R. J. (2003). Distinct spatial frequency sensitivities for processing faces and emotional expressions. *Nat. Neurosci.* 6 624–631. 10.1038/nn1057 12740580

[B97] Wieser M. J. Pauli P. Reicherts P. Mühlberger A. (2010). Don’t look at me in anger! Enhanced processing of angry faces in anticipation of public speaking. *Psychophysiology* 47 271–280. 10.1111/j.1469-8986.2009.00938.x 20030758

[B98] Zaki J. Ochsner K. (2009). The need for a cognitive neuroscience of naturalistic social cognition. *Ann. N. Y. Acad. Sci.* 1167 16–30. 10.1111/j.1749-6632.2009.04601.x 19580548 PMC2897139

[B99] Zhang D. Wang L. Luo Y. Luo Y. (2012). Individual differences in detecting rapidly presented fearful faces. *PLoS one* 7:e0049517. 10.1371/journal.pone.0049517 23166693 PMC3498139

[B100] Zhang Y. Chen Y. Bressler S. L. Ding M. (2008). Response preparation and inhibition: the role of the cortical sensorimotor beta rhythm. *Neuroscience* 156 238–246. 10.1016/j.neuroscience.2008.06.061 18674598 PMC2684699

[B101] Zhao F. Kang H. You L. Rastogi P. Venkatesh D. Chandra M. (2014). Neuropsychological deficits in temporal lobe epilepsy: a comprehensive review. *Ann. Indian Acad. Neurol.* 17 374–382. 10.4103/0972-2327.144003 25506156 PMC4251008

[B102] Zion-Golumbic E. Kutas M. Bentin S. (2010). Neural dynamics associated with semantic and episodic memory for faces: evidence from multiple frequency bands. *J. Cogn. Neurosci.* 22 263–277. 10.1162/jocn.2009.21251 19400676 PMC2807899

